# Serine 129 Phosphorylation of *α*-Synuclein Cross-Links with Tissue Transglutaminase to Form Lewy Body-Like Inclusion Bodies

**DOI:** 10.5402/2011/732879

**Published:** 2011-04-11

**Authors:** Wei Bi, GuoHua Zhang, Yuanlin Sun, Lihong Zhu, Chuanming Wang, Yanran Liang, Qiaoyun Shi, Enxiang Tao

**Affiliations:** ^1^Department of Neurology, Sun Yat-sen Memorial Hospital, Sun Yat-sen University, Guangzhou 510120, China; ^2^Dpartment of Neurology, The First People's Hospital of Foshan, Foshan 528000, China; ^3^Department of Pathophysiology, School of Medicine, JiNan University, Guangzhou 510632, China; ^4^Center for Inherited Cardiovascular Disease, Division of Cardiovascular Medicine, Stanford University School of Medicine, Stanford, CA 94304, USA

## Abstract

Intraneuronal depositions of *α*-synuclein have been implicated in the pathogenesis of Parkinsons's disease (PD). Previous reports have identified the crosslinking between *α*-synuclein and tTG (tissue transglutaminase) in both PD patients and the cellular model. However, no researches have been conducted to further investigate their interaction in physiological conditions. To address this question, we generated the SH-SY5Y cell line which stably expressed the wild-type or mutant (Ser129Ala) *α*-synuclein. After the treatment with okadaic acid, *α*-synuclein started forming aggregates upon the activation of tTG. Coimmunoprecipitation assays revealed a decreased interaction of the mutant *α*-synuclein S129A with tTG compared with the wild-type *α*-synuclein. Cells expressing the wild-type *α*-synuclein showed increased eosinophilic cytoplasmic inclusion bodies that resembled Lewy bodies compared with the mutant. Double immunofluorescence staining confirmed the colocalization of the phosphorylated *α*-synuclein and the tTG in the cells. The S129A mutant demonstrated a lesser degree of colocalization than the wild type.

## 1. Introduction

Parkinson's disease (PD) is a common neurodegenerative disorder. Hallmarks of PD are the degeneration of dopaminergic neurons in the substantia nigra pars compacta (SNpc) and the presence of cytoplasmic inclusions known as Lewy bodies (LBs) in surviving neurons [1]. Although it is still under debate whether *α*-synuclein, the major component of Lewy bodies, plays a key role in the death of dopamine neurons, there is growing evidence suggesting its involvement in PD pathogenesis [2]. The well-accepted hypothesis proposes that the functional *α*-synuclein undergoes conformational change in pathological conditions which prevents the degradation of the abnormal proteins. As a consequence, these abnormal proteins aggregate to form cytotoxic Lewy bodies [3]. 

It has been shown that there is increased serine phosphorylation at SNpc in both familial and sporadic PD patients. Four serine phosphorylation sites have been identified in *α*-synuclein, Ser9, Ser42, Ser87, and Ser129. Ser129 is the most common one. Neurotoxic agents such as paraquat, and rotenone, 1-methyl-4-phenyl-1,2,3,6-tetrahydropyridine (MPTP) can cause serine phosphorylation of *α*-synuclein at position 129 [4]. Further studies showed that serine phosphorylation at position 129 promoted the formation of a-synuclein aggregates in the cytoplasm [5]. Despite the above evidence, there are reports demonstrating no alteration or even the prevention of a-synuclein aggregation after phosphorylation [6, 7]. The controversy might be explained by the different models used in the investigations, for example, some agents may function as the catalyzer or the crosslinker to enhance the aggregation of the *α*-synuclein.

Tissue transglutaminase (tTG) is a key member of transglutaminases family. It is predominantly expressed in the central nervous system and has functions in neural development, neural regeneration, and others [8, 9]. tTG mediates the posttranslational modification of proteins including the crosslinking and the polypeptide formation. This reaction is calcium dependent and inhibited by guanosine triphosphte (GTP) [10, 11]. tTG induces the protein-protein crosslinking via transaminations, the formation of amide bonds between the glutamine side chains and the *ε*-amino groups of lysine residues [12, 13]. It is proposed that the protein-protein crosslinking catalyzed by the tTG plays a causative role in various neurodegenerative diseases [14]. The crosslinking between *α*-synuclein and tTG had been reported in PD patients and the cellular models that were cotransfected with *α*-synuclein and tTG. Further investigations are needed to address their participation in cell physiology, along with their interaction in pathophysiology. 

In this study, we investigated the role of serine 129 phosphorylation of *α*-synuclein in the formation of eosinophilic cytoplasmic inclusions in vitro. The mutant *α*-synuclein S129A showed significantly reduced cytoplasmic eosinophilic inclusion bodies compared with the wild-type *α*-synuclein. When *α*-synuclein is immunocoprecipited with the tTG, the mutant presented with decreased protein aggregation. We also discovered that the tTG phosphorylated *α*-synuclein at Ser129 and activated the aggregation of the abnormal proteins to form inclusion bodies. Our results strongly supported the involvement of the *α*-synuclein in PD pathogenesis.

## 2. Material and Methods

### 2.1. Chemicals and Reagents

Cell culture media, LipofectAMINE Plus reagent, and Agarose beads were purchased from Invitrogen (Carlsbad, CA). Anti-*α*-synuclein monoclonal antibody was from BD Biosciences (PaloAlto, CA), antiphospho-S129 *α*-synuclein monoclonal antibody was from Woko (Osaka, Japan), antitissue transglutaminase polyclonal antibody was from Abcam (London, UK), antitissue transglutaminase monoclonal antibody was from Thermo (Thermo, USA), horseradish peroxidase- (HRP-) conjugated *β*-actin was from kangchen (Shanghai, China), Cyanine 3- (Cy3-) conjugated goat antimouse IgG, fluorescein- (FITC-) conjugated goat antirabbit IgG, and all-trans retinoic acid and monodactyl acid were from Sigma (St. Louis, MO, USA). Okadaic acid was from Alexis (Plymouth, USA), HE staining kit was from Applygen (Beijing, China), G418 was from GIBCO (Grand Island, USA), and HRP-conjugated antimouse and antirabbit secondary antibodies were purchased from Boster (Wuhan, China).

### 2.2. Plasmid Construction, Site-Directed Mutagenesis, and Transfection

The wild-type *α*-synuclein expressing plasmid was a generous gift from Dr. Raohua Li. The Quickchange Lighting Site-Directed Mutagenesis Kit was used to generate the mutant S129A following the manufactory protocol. The wild type and the mutant were confirmed by the sequencing. The pcDNA3.1(+)/*α*-ynuclein or the pcDNA3.1(+)/S129A*α*-synuclein expressing plasmids were added to lipofectamine 2000 to transfect human neuroblastoma cells SH-SY5Y to produce their stable expression cell lines. The transfection was performed following the manufactory's instruction. Western blot was conducted to confirm the expression of the wild-type and the mutant *α*-synuclein. 

### 2.3. Cell Culture and tTG Production

SH-SY5Y cells were Cultured in Dulbeeeo's Modified Eagle's Medium (DMEM) that was supplemented with 10% fetal serum and 300 mg/mL G418, at 37°C with 5% CO_2_. To produce tTG in the system, cells were cultivated in the above medium for 7 days except that 2% fetal serum and 20 umol/L retinoic acid were added (Zhang et al., 1998). All experiments were carried out in subconfluent cell cultures. 

### 2.4. Hematoxylin and Eosin Staining, Double Immunofluorescence

Hematoxylin and eosin (H&E) staining was performed following the manufactory instruction. Double immunofluorescence was conducted as described previously (Tanaka et al., 2001). In short, cells were washed with 1 × phosphate-buffered saline (PBS), fixed with 4% paraformaldehyde for 15 min, and permeabilized with 0.2% Triton X-100 for 10 min, followed by incubation with the primary antibodies of anti-*α*-ynuclein (1 : 100), antitissue transglutaminase (1 : 100), along with the secondary antibodies of Cy3-conjugated antimouse (1 : 200) or FITC-conjugated antirabbit (1 : 100) for 2 hours at room temperature. Cells were analyzed under the fluorescence microscopy (Nikon, Japan). Fluorescence intensity was measured to access the formation of inclusion bodies. The fluorescence positive cells were counted in six different microscopic fields with 1000 cells analyzed in each experimental condition. 

### 2.5. Western Blot

Western blot was performed as the following. Cells were washed with ice-cold PBS and lysed with 100 *μ*L (RIPA lysis buffer (10%Tris-HCl, 20% sodium deoxycholate, 10% glycerol) containing protease inhibitors. The sample was incubated at 4°C for 30 minutes and followed by centrifugation at 10,000 rpm for 10 min. The supernatant was collected, and the protein concentration was measured using bicinchoninic acid- (BCA-) 100 Protein Quantitative Analysis Kit (Shenergy Biocolor BioScicnce & Technology Company, China). 25 *μ*g of the protein sample was loaded for western blot analysis. After gel electrophoresis, protein samples were transferred onto a polyvinylidene difluoride (PVDF) membrane, blocked by 5% nonfat dry milk in 1 × Tris buffered saline containing 0.1% Tween-20 for 2 h, and incubated with the primary antibodies of anti-*α*-synuclein (1 : 1000), at 4°C overnight. The membrane was washed and the incubated with horseradish peroxidase.

 (HRP)-conjugated goat antimouse or antirabbit IgG (1 : 2000) have been incubated for 1 h at room temperature, and developed with an ECL-plus chemiluminescencekit for 1–5 min and exposed to a X-film. To demonstrate the equal loading of the protein in each lane, the membranes were stripped (100 mM 2-mercaptoethanol, 2%SDS, 62.5 mMTris–HCl, pH 6.7) and reprobed with HRP-conjugated *β*-actin (1 : 5000 dilution) antibody, followed by the exposure to an X-film. The images were captured using an Imager gel documentation system, and the band intensities were evaluated by the densitometric analysis using the imager software. 

## 3. Statistical Analysis

All results were presented as Mean ± SD based on at least three independent experiments performed in duplicates. Overall differences among groups were determined by analysis of variance (ANOVA). A posteriori pairwise differences were determined using the Student-Newman-Keuls *q*-test. Data were analyzed by SPSS13.0 software. *P* < .05 was considered statistically significant.

## 4. Results

### 4.1. Generation of the Wild-Type and the Mutant *α*-Synuclein (S129A) Expressing Cell Lines

**Figure 1:**
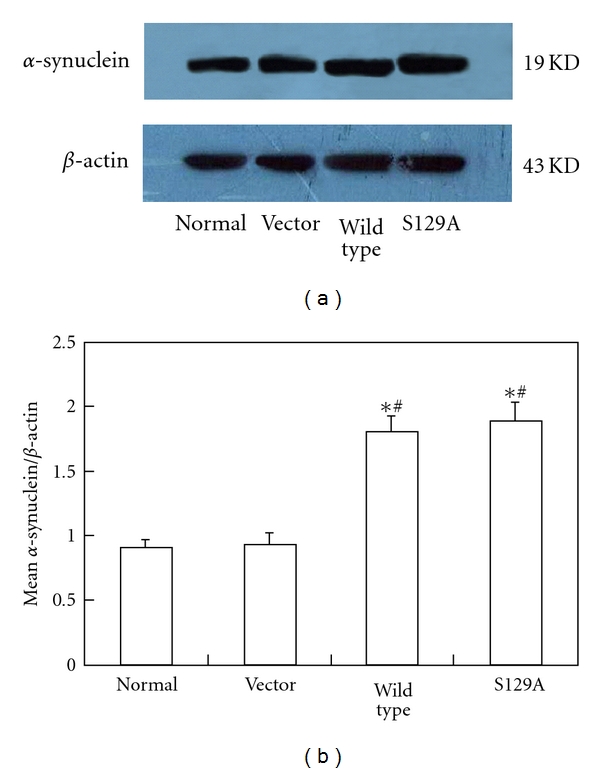
Stable expression of the wild and mutant *α*-synuclein in vitro. Immunoblots of *α*-synuclein and *β*-actin in SH-SY5Y cells without transfection (Lane 1), transfected with empty vector (Lane 2), or vectors encoding the wild-type (Lane 3) or mutant (Lane 4) *α*-synuclein. Cell lysates were analyzed by immunoblotting using anti-*α*-synuclein antibody. *β*-actin was used as a loading control. The wild-type *α*-synuclein and the S129A *α*-synuclein protein in transfection groups were obviously higher than that in nontransfected and empty plasmid transfected groups.

Western blot results showed that the wild-type *α*-synuclein and the S129A *α*-synuclein protein in transfection groups were obviously higher than that in nontransfected and empty plasmid transfectied groups (Figure 1). These suggested that the wild-type *α*-synuclein and the mutant S129A *α*-synuclein were induced into target cells and expressed successfully.

### 4.2. Downregulation of the Cytoplasmic Colocalization of tTG and *α*-Synuclein in the Mutant S129A

**Figure 2:**
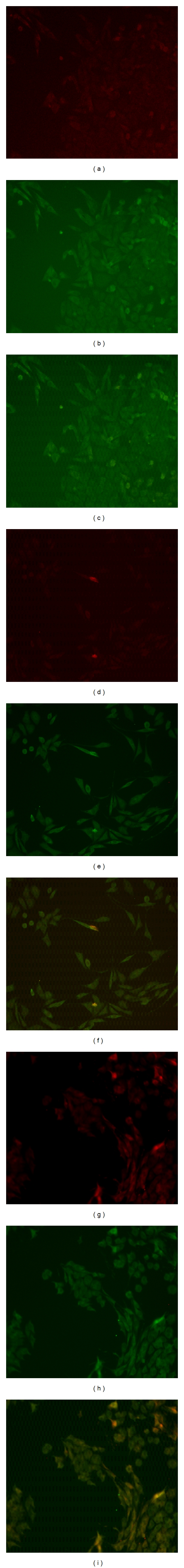
Downregulation of the colocalization of the mutant *α*-synuclein (S129A) and tTG. Immunofluorescence of P129 *α*-synuclein (red) and tTG (green) in three cell lines (×400). Cells were transfected with empty vectors (a–c), or vectors encoding the wild-type (d–f) or mutant (g–i) *α*-synuclein; cells were double-labeled with antibodies against P129 *α*-synuclein ((a), (d), (g), Cy3-conjugated) and tTG ((b), (e), (h), FITC-conjugated) to detect the colocalization of *α*-synuclein and tTG. (c, f, i) are merged images (yellow indicates colocation) of cell bodies; Cells were incubated in the presence of 20 *μ*M retinoic acid (RA) for 7 days, then treated by 20 nM okadaic acid for 24 h. The mutant S129A demonstrated the downregulated colocalization compared with the wild-type and the control cells.

The serine phosphorylated *α*-synuclein colocalized with the tTG in the cytoplasma predominantly. Intranuclear colocalization was identified but less common. The mutant S129A demonstrated a reduction in colocalization compared with the wild-type and the control cells (Figure 2).

### 4.3. Decrease in *α*-Synuclein-tTG Crosslinks in the Mutant S129A

**Figure 3:**
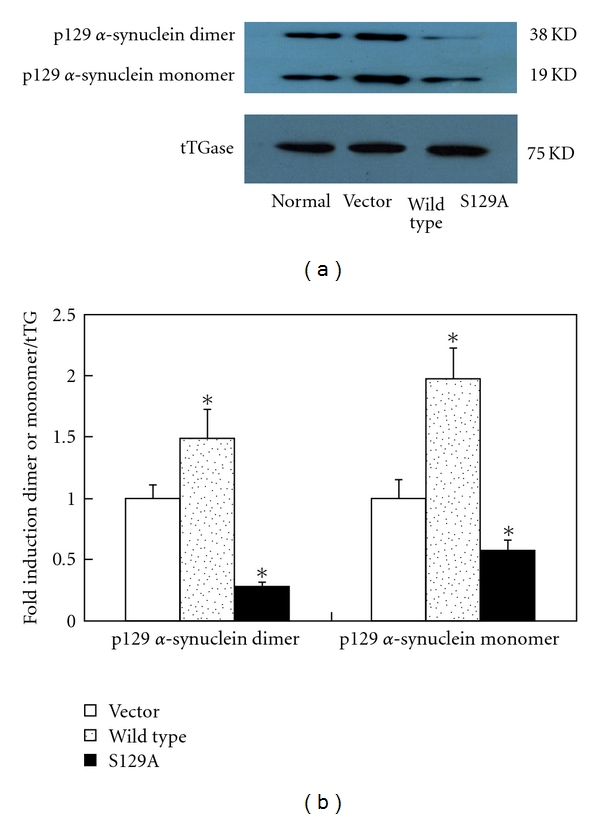
Decreased crosslinking between tTG and the mutant *α*-synuclein S129A. Cells were transfected with empty vectors, vectors encoding the wild-type or the mutant *α*-synuclein S129A. They were treated with 20 *μ*M retinoic acid for 7 days and incubated for 24 h in the presence of 20 nM okadaic acid to generate tTG in the system. Immunoprecipitation was performed to access the interaction between tTG and *α*-synuclein. The mutant S129A showed significant reduction in the crosslinking between the tTG and the *α*-synuclein monomer and dimer. *P < .01 compared with cells transfected with empty vectors.

Both serine phosphorylated *α*-synuclein dimers and monomers interacted with tTGs. After OA treatment, the mutant S129A demonstrated significantly decreased crosslinks between the *α*-synuclein and the tTG compared with the wild type (*P* < .01). The crosslinks were reduced to lower than that of the baseline control cells transfected with empty vectors (Figure 3).

### 4.4. Inhibition of Lewy Body-Like Inclusion Body Formation and Apoptotic Cell Death in the Mutant S129A

**Figure 4:**
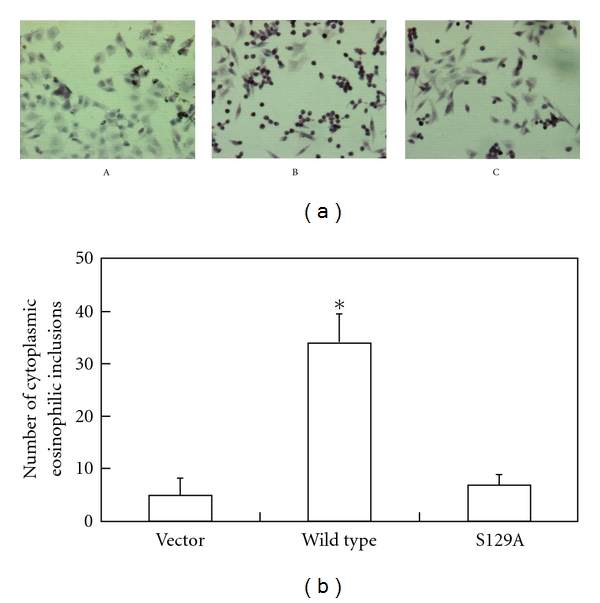
Reduced cytoplasmic inclusion body formation and apoptotic cell death in the *α*-synuclein mutant S129A. Cells were transfected with empty vectors, vectors encoding the wild-type or the mutant *α*-synuclein S129A. They were treated with 20 *μ*M retinoic acid for 7 days followed by incubation in 20 nM okadaic acid for 24 h (A, B, C). The mutant S129A demonstrated significantly decreased cytoplasmic inclusion body formation compared with the cells transfected with the wild-type *α*-synuclein.

H&E stain showed that 24 h posttreatment with OA, there was down-regulation of eosinophilic inclusion body formation in the mutant S129A compared with the wild type (*P* < .01). The mutant S129A demonstrated significantly decreased cytoplasmic inclusion body formation compared with the cells transfected with the wild-type *α*-synuclein (Figure 4).

## 5. Discussion

The presence of insoluble Lewy bodies in the brain of PD patients is the pathological hallmark of the disease. It has been identified that the *α*-synuclein and tTG are the two major components of the Lewy bodies. Although it remains inconclusive about the role of *α*-synuclein in the pathogenesis of PD, in vitro and in vivo studies have shown that *α*-synuclein is a cellular substrate of tTG [15–17]. In a cell model, cos-7 cells were transfected with the wild-type *α*-synuclein plasmid in the absence or presence of tissue transglutaminase. Cotransfection with the tTGase expressing plasmids induced the formation of insoluble *α*-synuclein aggregates. The aggregation was tTGase dose dependent [18]. In this study, we further investigated the interaction between *α*-synuclein and tTG in vitro via the upregulation of tTG using retinoic acid followed by Monodansyl acid addition to block its further production [19]. Our results showed that the suppression of the tTG decreased cytoplasmic eosinophilic inclusion formation when treated with okadaic acid. The inclusion formation was significantly inhibited in the *α*-synuclein mutant S129A. Our results indicated that the crosslinking of *α*-synuclein and tTG regulated the formation of cytoplasmic Lewy body-like inclusion bodies. 

*α*-synuclein is modulated by several posttranslational modifications [20]. The serine 129 phosphorylation is one of the most important posttranslational modifications [21, 22]. It has been reported that serine 129 phosphorylation of *α*-synuclein contributes to the development of PD [21, 23]. Several protein kinases, such as CK1, CK2, and a family of G-protein-coupled receptor kinases (GRKs), have been found to phosphorylate alpha-synuclein [24, 25]. However, it is not clear whether serine 129 phosphorylation plays an essential role in Lewy body formation. It was reported that the blockage of of serine 129 phosphorylation increased inclusion formation in *α*-synuclein transgenic flies [26]. In this study, we investigated the serine phosphorylation and its regulation of inclusion body formation using a mammalian cell model. We discovered that the mutation S129A prevented the phosphorylation of *α*-synuclein, therfore suppressed its cytoplasmic aggregation (Figure 4). Earlier studies found that the activation of tTG resulted in the formation of insoluble aggregates of wild-type *α*-synuclein [22]. However, There were concerns that the finding might not be physiologically relevant by the transient expression of *α*-synuclein in the investigation. Furthermore, there is discrepancy that investigations using stable expression cells found no aggregation of *α*-synuclein [24, 26]. This phenomenon might be explained due to the relatively low expression levels of *α*-synuclein in stable cell lines, suggesting that expression levels of *α*-synuclein are a critical factor for the aggregate formation of *α*-synuclein [27]. 

## 6. Conclusions

We demonstrated that Ser129 phosphorylation was required for the crosslinking of *α*-synuclein and tTG. Their interaction induced the formation of cytoplasmic Lewy body-like inclusion bodies. Our results strongly support that *α*-synuclein, tTG, and their interaction contribute to the development of Parkinson's disease.
